# SARS-CoV-2 resistance analyses from the Phase 3 PINETREE study of remdesivir treatment in nonhospitalized participants

**DOI:** 10.1128/aac.01238-24

**Published:** 2024-12-19

**Authors:** Lauren Rodriguez, Hery W. Lee, Jiani Li, Ross Martin, Dong Han, Simin Xu, Jasmine Moshiri, Nadine Peinovich, Gregory Camus, Jason K. Perry, Robert H. Hyland, Danielle P. Porter, Mazin Abdelghany, Matthias Götte, Charlotte Hedskog

**Affiliations:** 1 Gilead Sciences, Inc. 2158 , Foster City, California, USA; 2 Department of Medical Microbiology and Immunology, University of Alberta 3158 , Edmonton, Alberta, Canada; Bill & Melinda Gates Medical Research Institute, Cambridge, Massachusetts, USA

**Keywords:** drug therapy

## Abstract

Remdesivir inhibits the SARS-CoV-2 RNA-dependent RNA polymerase (RdRp; Nsp12). Here, we conducted viral resistance analyses from the Phase 3 PINETREE trial of remdesivir in nonhospitalized participants at risk of severe COVID-19. Nasopharyngeal swabs (collected at baseline [Day 1], Days 2, 3, 7, and 14) were eligible for analysis if their viral load was above the lower limit of quantification for the RT-qPCR assay (2228 copies/mL). The SARS-CoV-2 genome was sequenced for all remdesivir participants and 50% of placebo participants (baseline, Days 3, 7, and 14) and for participants who progressed to COVID-19-related hospitalization or all-cause death (all time points). Emergent substitutions in Nsp12 and other replication complex proteins were phenotyped using site-directed mutagenesis in a SARS-CoV-2 subgenomic replicon system. Overall, emergent Nsp12 substitutions were detected in 8/115 (7.0%) remdesivir participants and 7/129 (5.4%) placebo participants (1 substitution overlap between groups). Based on a structural analysis, none of the emergent Nsp12 substitutions were in direct contact with the incoming nucleoside triphosphate substrate, the RNA, or the RNA template 5′ overhang. One substitution (A376V) showed reduced susceptibility to remdesivir (12.6-fold change in remdesivir half-maximal concentration [EC_50_]); it also showed reduced fitness when introduced in the SARS-CoV-2 replicon and virus *in vitro*. Other substitutions had <1.1-fold change in remdesivir EC_50_. None of the emergent substitutions in Nsp8, Nsp10, Nsp13, or Nsp14 (remdesivir, 10/115 [8.7%]; placebo, 10/129 [7.8%]) showed reduced remdesivir susceptibility. In conclusion, emergent substitutions in the SARS-CoV-2 RdRp complex with reduced remdesivir susceptibility were uncommon, indicating a high barrier to remdesivir resistance.

**CLINICAL TRIALS:**

This study is registered with ClinicalTrials.gov as NCT04501952.

## INTRODUCTION

Remdesivir (GS-5734; Veklury) is a nucleotide analog prodrug that is intracellularly metabolized into an active analog of adenosine triphosphate and inhibits the RNA-dependent RNA polymerase (RdRp) complex of multiple viruses, including members of the *Coronaviridae*, *Flaviviridae*, *Filoviridae*, and *Pneumoviridae* families (1–4). RNA viruses, such as SARS-CoV-2, have high mutation rates (5), which enable adaptation to changes in the environment, immune escape, and resistance to monoclonal antibodies (6, 7). Additionally, several SARS-CoV-2 mutations have been reported in patients following treatment with monoclonal antibodies (8–11). Emergence of substitutions with reduced susceptibility following antiviral treatments for COVID-19 is rare (12–15).

The RdRp complex of SARS-CoV-2 has shown high genetic stability, making it a valuable target for antiviral treatment (16). The target of remdesivir is the nonstructural protein Nsp12, which contains the RdRp active site (17–19). Studies to date have shown that the most commonly observed Nsp12 amino acid substitution in SARS-CoV-2 variants, including Delta and Omicron, is P323L (>90% of sequences) (20, 21), which is not associated with reduced susceptibility to remdesivir (22).

*In vitro* resistance selection experiments have identified a number of emergent substitutions in Nsp12 (including V166A, V166L, N198S, S759A, C799F, C799R, V792I, E802A, and E802D), which conferred minimal to low-level reduced susceptibility to remdesivir (half-maximal effective concentration [EC_50_] increases of 2.1- to 10.4-fold), but these may be associated with a fitness cost (23–27). A case study has reported a mutation in Nsp12 (E802D), which was associated with an increased remdesivir EC_50_ and a transient virologic response, although the patient’s symptoms resolved (27). Notably, remdesivir maintains full activity against SARS-CoV-2 variants, including Alpha, Beta, Gamma, Delta, Epsilon, Kappa, Iota, Lambda, Zeta, and Omicron (22, 28–30).

**Fig 1:**
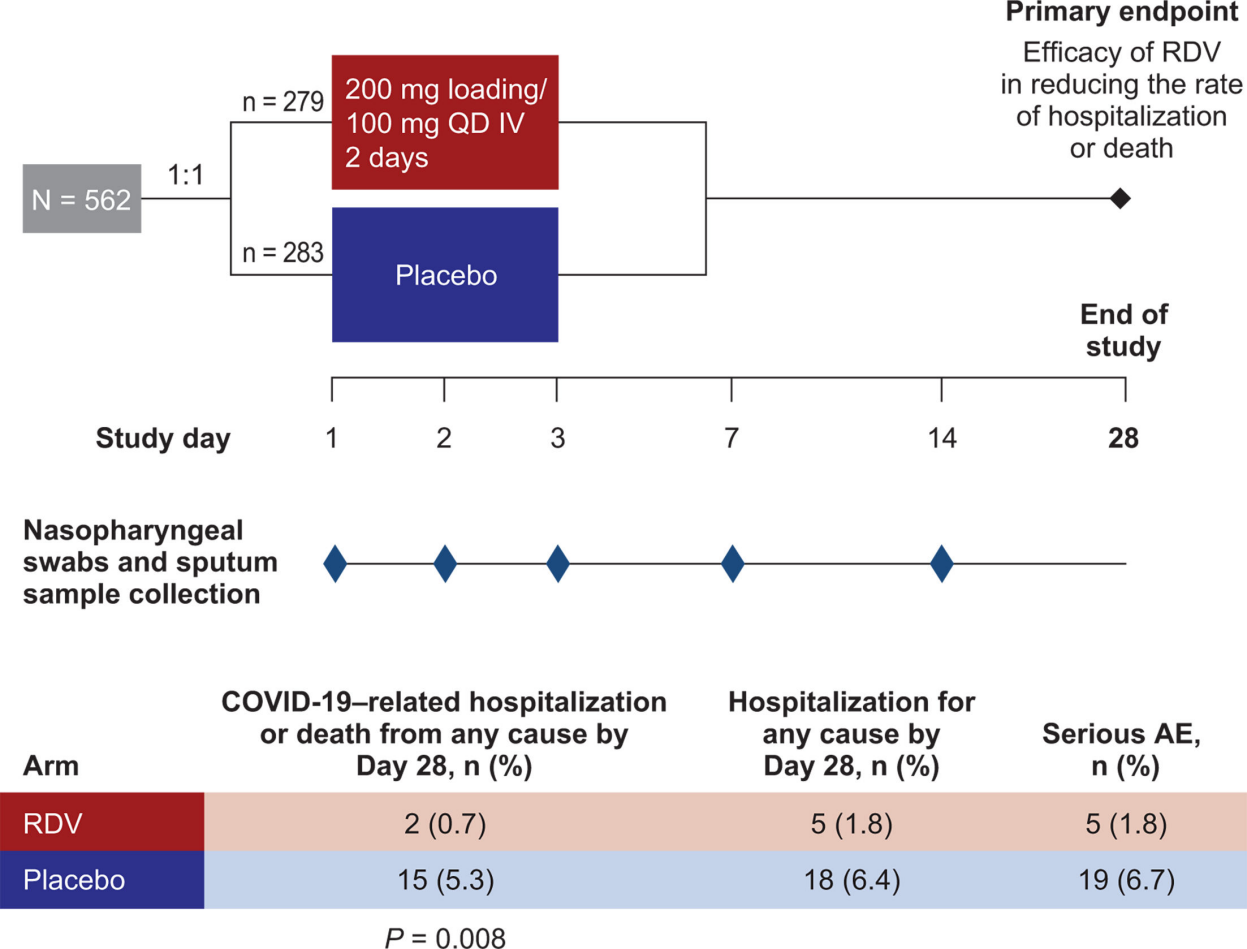
PINETREE study design. AE, adverse event; IV, intravenous; QD, once daily; RDV, remdesivir.

Remdesivir is approved for the treatment of COVID-19 in hospitalized patients and nonhospitalized patients at high risk of progression to severe disease in the United States, the European Union, Japan, and in other countries for pediatric and adult patients (31, 32). A number of clinical studies have demonstrated the efficacy of remdesivir in shortening the time to recovery and improving clinical status as compared with the standard of care in hospitalized patients with COVID-19 (33, 34). In the pivotal Phase 3 PINETREE study, the risk of hospitalization or all-cause death by Day 28 was reduced by 87% with remdesivir compared with placebo in outpatients at high risk of progression to severe COVID-19 (age ≥60 years, obesity or selected medical comorbidities; Fig. 1) (35).

The present analyses assessed whether amino acid substitutions in Nsp12 and other components of the RdRp complex emerged during and after remdesivir treatment in the viral genome of PINETREE study participants and evaluated whether any emergent substitutions conferred reduced susceptibility to remdesivir.

## RESULTS AND DISCUSSION

### Participants

From a total of 562 participants treated in the PINETREE study, 281 participants met the criteria for SARS-CoV-2 sequencing, i.e., viral load >2228 copies/mL at baseline (130 participants in the remdesivir group and 151 participants in the placebo group). In the remdesivir group, baseline sequencing was successful in 119/130 (91.5%) participants, and postbaseline sequencing was successful in 122/130 (93.8%; Table 1). In the placebo group, sequencing was successful in 138/151 (91.4%) and 137/151 (90.7%) participants at baseline and postbaseline, respectively.

**TABLE 1:** SARS-CoV-2 sequencing data in the full analysis set^*a*^

Treated, *n*/*N* (%)	Remdesivir (*N* = 279)	Placebo (*N* = 283)	Total (*N* = 562)
Met resistance analysis criteria and sequencing attempted	130/279 (46.6)	151/283 (53.4)	281/562 (50.0)
Sequencing data available			
Baseline	119/130 (91.5)	138/151 (91.4)	257/281 (91.5)
Postbaseline	122/130 (93.8)	137/151 (90.7)	259/281 (92.2)
Baseline and postbaseline	115/130 (88.5)	129/151 (85.4)	244/281 (86.8)

^*a*^ The full analysis set included all participants who were randomized into the study and received ≥1 dose of study treatment. Baseline amino acid substitutions in Nsp12 were compared with the reference sequence (Wuhan-Hu-1, NC_045512.2). Postbaseline sequences were compared with baseline sequences for each individual participant for Nsp8, Nsp10, Nsp12, Nsp13, and Nsp14.

### Baseline virologic analyses

Of the 257 participants with baseline sequencing data available, the most common SARS-CoV-2 lineage detected at baseline was B.1.2 (78 participants; 30.4%), followed by B.1.1.7 (Alpha; 48 participants; 18.7%), B.1.429 (Epsilon; 23 participants; 8.9%) and B.1.243 (10 participants; 3.9%; Table S1). A total of 11 substitutions in Nsp12 were detected in two or more participants; the most common was P323L, which was present in all but one participant. This is consistent with current known circulating SARS-CoV-2 variants (20, 21). Phenotyping of P323L has shown no loss of susceptibility to remdesivir (22), and none of the remaining Nsp12 substitutions observed at baseline have been associated with reduced susceptibility to remdesivir.

### Emergent amino acid substitutions

Fifteen of the 244 participants with both baseline and postbaseline sequencing data had emergent substitutions in Nsp12 (Table S2). The proportion of participants with emergent Nsp12 substitutions was comparable for the remdesivir and placebo groups (8/115 [7.0%] and 7/129 [5.4%], respectively). A total of seven emergent Nsp12 substitutions were detected in the remdesivir group but not in the placebo group. The F694Y emergent substitution was observed in both the remdesivir (one participant) and placebo (two participants) groups. All emergent substitutions observed in the remdesivir group were found in one participant each. However, one participant in the placebo group had four emergent substitutions: E144G, D155H, R631G, and S835L. For the substitutions occurring in Nsp12, only P232L (Day 2) and A634S (Day 3) emerged during remdesivir treatment, whereas the other substitutions were detected at 4 to 11 days after remdesivir cessation. Notably, these were different substitutions from those observed in the ACTT-1 and SIMPLE studies (14, 15).

Virologic analysis from the ACTT-1 trial in hospitalized patients with COVID-19 also showed a similar rate of emergent Nsp12 substitutions in those treated with remdesivir compared with placebo (39% vs 40%) (14). This supports the hypothesis that the low fidelity of RdRp, including nucleotide misincorporation and erroneous template switching, may underlie SARS-CoV-2 variation rather than as a result of selective pressure from remdesivir treatment (36, 37).

Emergent substitutions in Nsp8, Nsp10, Nsp13, or Nsp14 were detected in 10/115 (8.7%) participants in the remdesivir group and 10/129 (7.8%) participants in the placebo group (Table S3). Three emergent substitutions in Nsp8 (remdesivir, one participant; placebo, two participants), four in Nsp10 (remdesivir, three participants; placebo, two participants), 13 in Nsp13 (remdesivir, six participants; placebo, four participants), and 10 in Nsp14 (remdesivir, two participants; placebo, six participants) were detected. One substitution (Nsp13 E201K) was detected in both the remdesivir and placebo groups. As of 7 May 2024, a range of 0 to 167,545 (0% to 1.02%) of 16,348,065 sequences with these substitutions were deposited in the GISAID database.

### Structural analysis of the emergent substitutions in participants treated with remdesivir

**Fig 2:**
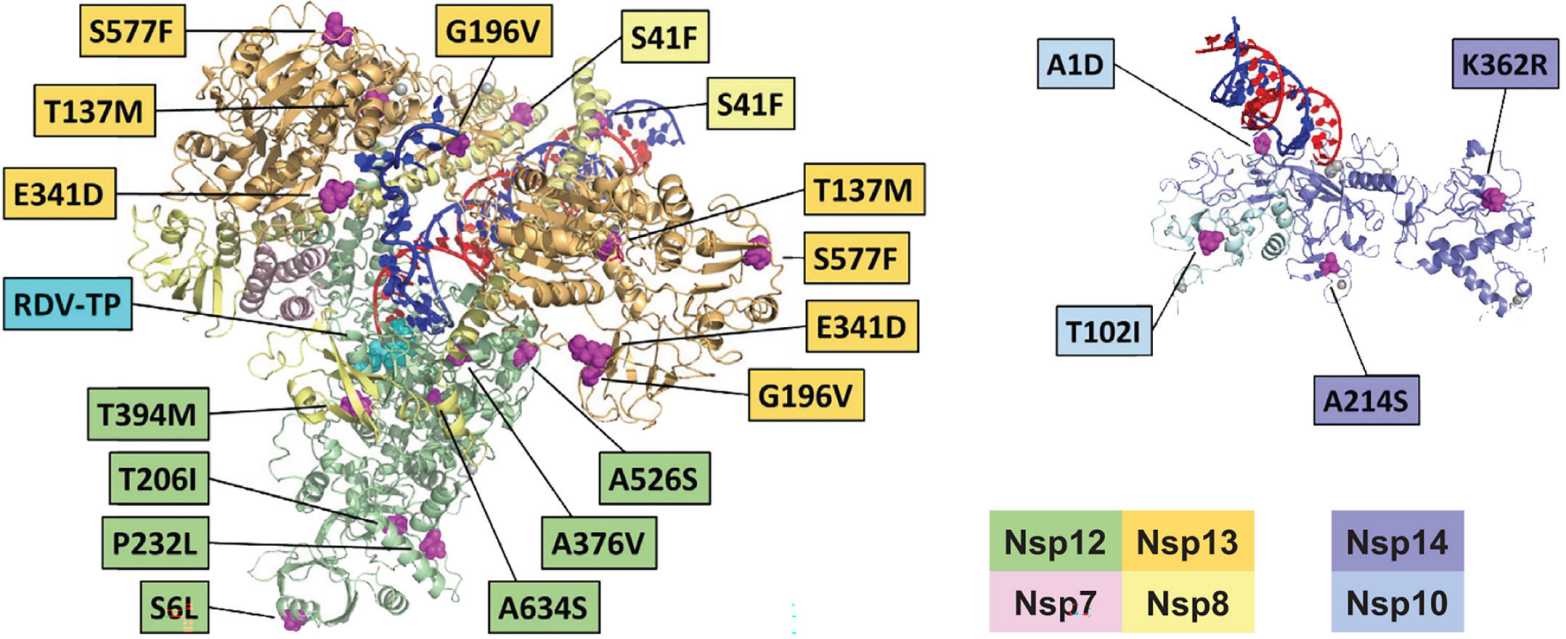
Cryo-EM map of observed amino acid substitutions within the SARS-CoV-2 RNA-dependent polymerase complex in the remdesivir group. Cryo-EM, cryo-electron microscopy; RDV-TP, triphosphate form of remdesivir. On the left is the complex formed from Nsp12, Nsp7, two subunits of Nsp8, and two subunits of Nsp13, modeled on a composite of two cryo-EM structures (7UO4 and 7RDX) (38). A pre-incorporated RDV-TP (colored in cyan) is seen in the polymerase active site, as captured in the 7UO4 structure. On the right is the complex formed by Nsp14 and Nsp10, modeled from the cryo-EM structure 7N0B. Substitutions are widely dispersed across the two complexes. Of note, the Nsp13 A598V substitution is not shown. It is located in the C-terminal tail of the protein, which is not resolved in observed structures, implying significant disorder.

Prior *in vitro* resistance selection studies that produced emergent substitutions in Nsp12, such as Nsp12 S759A, exhibited a biochemical resistance phenotype (e.g., a 10-fold decreased preference for the remdesivir-triphosphate) (23). Herein, a structural analysis found that none of the Nsp12 substitutions detected in the remdesivir group (S6L, T206I, P232L, A376V, T394M, A526S, and A634S) were in direct contact with the incoming nucleoside triphosphate substrate or the RNA (Fig. 2). A376V was located close to the template 5′ overhang. Overall, substitutions in Nsp8, Nsp10, Nsp13, and Nsp14 were sporadically dispersed across the proteins rather than clustered in particular areas, suggesting largely random mutation patterns as opposed to mutations induced by the mechanism of action of remdesivir.

Remdesivir has been shown to inhibit replication via a delayed chain termination following incorporation of its triphosphate form (18). Additionally, remdesivir has been shown to have a template-dependent mechanism, in which the nucleoside triphosphate metabolite of remdesivir incorporates into viral RNA, but that RNA in turn cannot function as an effective template (39). Mutations that affect the positioning of the template in the polymerase active site have the potential to modulate that activity. More generally, the fitness of the polymerase may also be impacted by mutations that affect template positioning (17, 23). Only Nsp10 A1D was in direct contact with the template RNA in the Nsp10-Nsp14 complex with dsRNA engaged with the exonuclease site, but any impact on the exonuclease proofreading capability of Nsp14 is unclear.

### Phenotypic analyses of emergent substitutions in the remdesivir-treated group

Phenotyping was conducted using site-directed mutants in a noninfectious replicon system to evaluate their effect on the *in vitro* susceptibility of SARS-CoV-2 to remdesivir. The majority of the successfully phenotyped emergent substitutions in Nsp8, Nsp10, Nsp12, Nsp13, and Nsp14 were similarly susceptible to remdesivir (≤2.3-fold change) as the wild-type reference replicon (mean EC_50_, 10.0 nM; Table 2), including the Nsp10 A1D substitution that was in direct contact with the template RNA. The replication capacity of the emergent substitutions compared to the wild-type control replicon ranged from 0.5% to 202.4%. The A1D substitution in Nsp10 showed no impact on the antiviral activity of remdesivir.

**TABLE 2:** Remdesivir EC_50_ fold change against and replication capacity of SARS-CoV-2 reference and mutant replicons^*a*^

Gene	Substitution	Mean (SD) remdesivir EC_50_, nM	EC_50_-fold change from WT	Replication capacity vs WT (%)^*b*^
	SH01 WT^*c*^	10.0 (2.2)	1.0	100.0
Nsp12	S759A/V792I (positive control)	99.1 (10.1)	9.9	9.2
Nsp8	S41F	7.8 (0.1)	0.8	152.8
Nsp10	A1D	23.2 (0.6)	2.3	1.2
Nsp10	T102I	18.3 (3.0)	1.8	72.4
Nsp12	S6L	NA^*d*^	NA^*d*^	NA^*d*^
Nsp12	T206I	8.4 (6.7)	0.8	0.5
Nsp12	P232L	7.3 (1.1)	0.7	0.6
Nsp12	A376V	126.4 (34.8)	12.6	2.1
Nsp12	T394M	12.0 (2.7)	1.2	35.7
Nsp12	A526S	10.9 (0.8)	1.1	53.6
Nsp12	A634S	14.0 (0.8)	1.4	202.4
Nsp13	T137M	10.7 (4.3)	1.1	1.4
Nsp13	G196V	NA^*d*^	NA^*d*^	NA^*d*^
Nsp13	E341D	8.5 (3.2)	0.9	12.5
Nsp13	S577F	NA^*d*^	NA^*d*^	NA^*d*^
Nsp13	A598V	10.7 (0.4)	1.1	50.1
Nsp14	A214S	14.5 (3.3)	1.4	28.7
Nsp14	K362R	11.5 (0.8)	1.4	16.1

^*a*^ EC_50_, half-maximal effective concentration; NA, not applicable; SD, standard deviation; WT, wild-type.

^*b*^ Replication capacity of the mutant replicon was calculated by dividing the maximum luciferase signal of the mutant replicon by the WT replicon at 48 hours postinfection.

^*c*^ SH01 is a WT reference SARS-CoV-2 replicon from clinical isolate from Shanghai (lineage B).

^*d*^ Mean remdesivir EC_50_, fold change from WT, and replication capacity could not be calculated due to lack of replication of the replicon.

**Fig 3:**
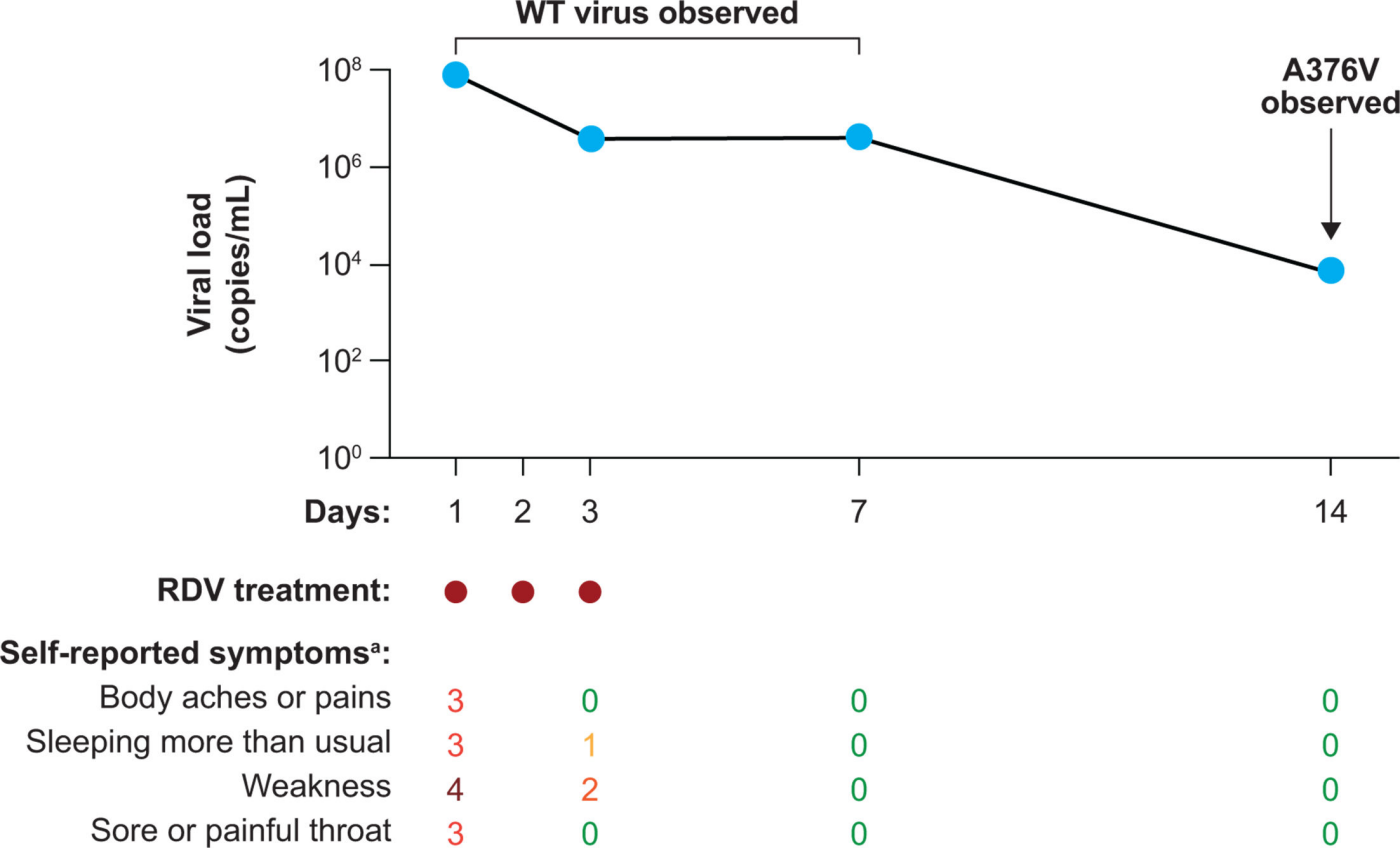
Viral load decrease and symptom alleviation in participants with Nsp12 substitution A376V. RDV, remdesivir; WT, wild-type. ^a^Self-reported symptom scale: (4) very much, (3) quite a bit, (2) somewhat, (1) a little bit, and (0) not at all.

One emergent Nsp12 substitution (A376V) associated with reduced susceptibility to remdesivir was detected in one participant (12.6-fold change in EC_50_) that was observed on Day 14 (11 days after remdesivir cessation). An analysis of publicly available sequences in the GISAID database has shown that the A376V substitution is rare (0.00002% [4/16,313,999] of SARS-CoV-2 sequences as of 6 May 2024). In the participant with the A376V substitution, SARS-CoV-2 viral load was reduced by 4 log_10_ copies/mL from baseline (81,136,769 copies/mL) to Day 14 (7535 copies/mL), and self-reported symptoms were alleviated by Day 7 (Fig. 3).

**Fig 4:**
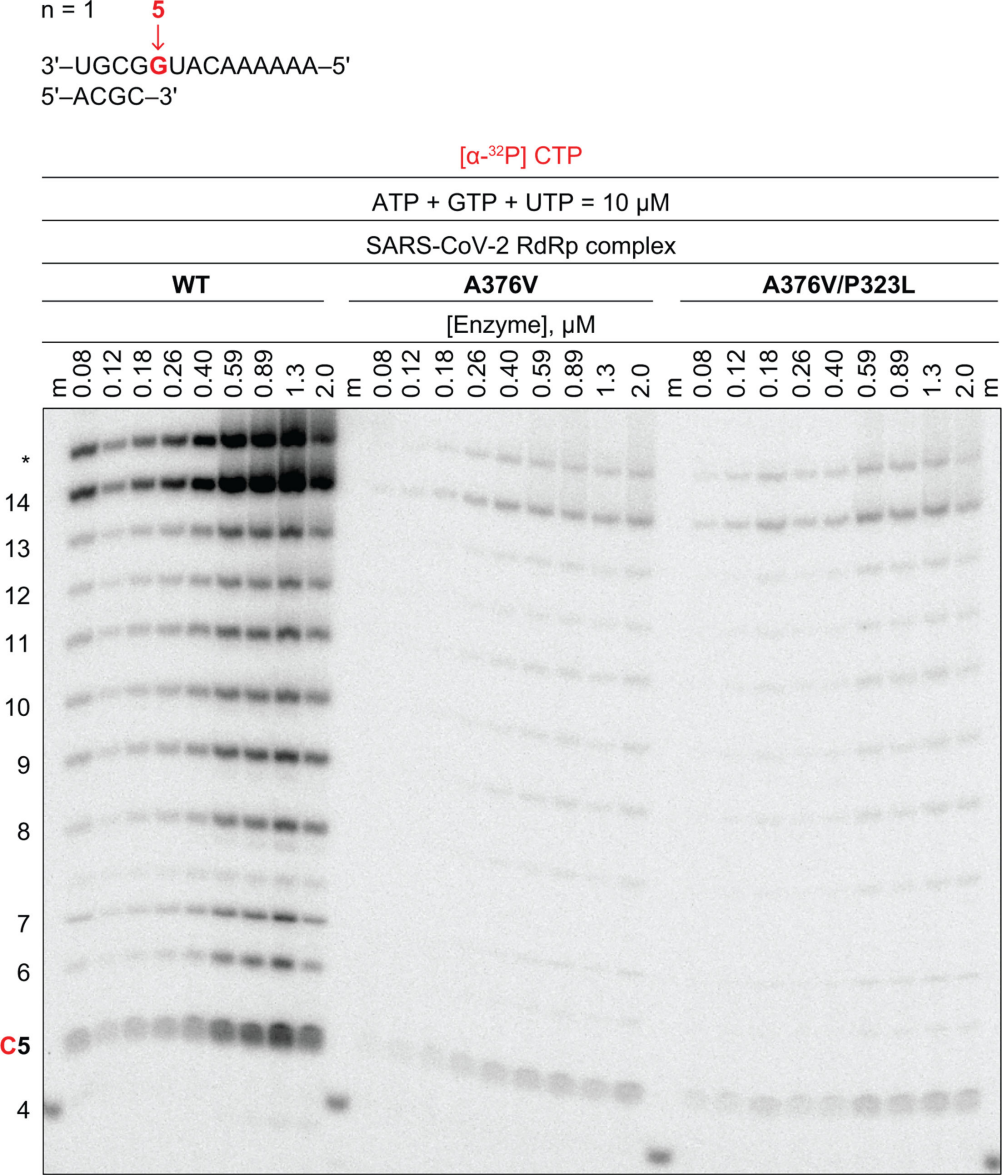
Enzymatic activity of A376V- and A376V/P323L-mutant RdRps on full-length RNA synthesis relative to WT. ATP, adenosine triphosphate; CTP, cytidine triphosphate; GTP, guanosine triphosphate; UTP, uridine triphosphate; RdRp, RNA-dependent RNA polymerase; WT, wild-type. RNA template and primer sequences used are shown at the top. RNA products synthesized by the WT, A376V-, and A376V/P323L-mutant RdRps. The reaction mixture contained [α-^32^P] CTP for detection, the indicated nucleotide-triphosphates for full-length RNA extension, 5 mM MgCl_2_ cofactor, and increasing concentrations of RdRps. The sizes of extended RNA products in nucleotides are shown on the left. m denotes a radioactively labeled, 4-nucleotide primer used as a size marker. C (red) indicates the position at which [α-^32^P] CTP is incorporated. *Indicates RNA products resulting from terminal transferase activity. Results are representative of a total of two replicates.

A SARS-CoV-2 replicon containing the A376V substitution in Nsp12 showed a replication capacity of only 2.1% relative to the wild-type control replicon (*P* <0.0001; Table 2). To compare the enzymatic activity of the mutant polymerase with that of the wild-type, the A376V substitution was introduced in Nsp12, the Nsp7-Nsp8-Nsp12 complex with the mutated Nsp12 protein was purified, and RNA extension was detected using radiolabeled nucleotides. Both the A376V- and A376V/P323L-mutant RdRp were severely compromised in their ability to promote RNA extension (Fig. 4).

Generation of Nsp12 A376V-mutant SARS-CoV-2 using an *in vitro* ligation-based reverse genetics system was not successful in three independent attempts, though SARS-CoV-2 carrying the wild-type amino acid at this position (376A) was repeatedly generated in parallel (Table S4). In two of three attempts, no viral RNA or cytopathic effect was detected in cells electroporated with full-length viral RNA encoding the Nsp12 A376V substitution. In a third attempt, full-length viral RNA encoding Nsp12 A376V reverted to wild-type Nsp12 sequence within 4 days postelectroporation. These results suggest a severe fitness cost of the Nsp12 A376V substitution to SARS-CoV-2 that does not support viral growth in cell culture. Overall, a resistant phenotype was not identified (data not shown). Previous *in vitro* resistance selection studies with remdesivir have shown that other Nsp12 substitutions, while increasing the EC_50_-fold change relative to wild-type SARS-CoV-2 in some instances, are also associated with a fitness cost (25, 26). The severely compromised activity of the mutant enzymes makes it difficult to establish a biochemical phenotype that would help to explain the observed resistance pattern.

### Conclusion

In this analysis, baseline and postbaseline sequencing data were obtained from participants in the pivotal Phase 3 PINETREE study of remdesivir in participants at high risk of progression to severe COVID-19. The proportion of emergent Nsp12 substitutions was comparable for the remdesivir and placebo groups. Notably, the Nsp12 P323L substitution was found in all but one participant at baseline and was not associated with reduced susceptibility to remdesivir (22). None of the Nsp12 substitutions detected in the remdesivir group were found to be in direct contact with the incoming nucleoside triphosphate substrate or the RNA. Of all the emergent Nsp12 substitutions detected in the remdesivir group, only the A376V substitution was associated with a reduced susceptibility to remdesivir. Notably, this alteration was associated with a substantial fitness cost to SARS-CoV-2. The participant with this substitution had an alleviation of baseline symptoms by Day 7 and a 4-log reduction in viral load by Day 14. Emergent substitutions in Nsp8, Nsp10, Nsp13, or Nsp14 were detected in 10 participants in the remdesivir group, but none were associated with reduced susceptibility to remdesivir. A structural analysis of the emergent substitutions across Nsp12, Nsp8, Nsp10, Nsp13, and Nsp14 suggests no pattern to the mutations that might be linked to remdesivir’s mechanism of action.

There are some limitations to this analysis. It was not possible to derive sequencing data for all participants due to insufficient viral load, a missing sample, or assay failure due to low viral load in 305/562 (54%) participants at baseline and 303/562 (54%) participants postbaseline. The study was conducted during the period of the SARS-CoV-2 Alpha variant predominance, and therefore the emergence of Nsp12 mutations associated with remdesivir treatment during the Delta and Omicron waves was not observed at the time of this study. However, *in vitro* studies have shown that remdesivir maintains activity against these variants (22, 28, 40).

In conclusion, emergent Nsp12 substitutions in participants from the PINETREE study were uncommon and indicate a high barrier to the development of remdesivir resistance in patients with COVID-19.

## MATERIALS AND METHODS

### Clinical study

Details of the double-blind, randomized, placebo-controlled PINETREE study (ClinicalTrials.gov identifier NCT04501952) have been published previously (35, 41). Participants aged ≥12 years with ≥1 preexisting risk factor for progression to severe COVID-19 (including selected comorbidities: hypertension, cardiovascular or cerebrovascular disease, diabetes, obesity, immune compromise, chronic mild-moderate kidney disease, chronic liver or lung disease, current cancer, or sickle cell disease) or aged ≥60 years were eligible to participate in the study. Baseline demographic information was collected, including sex; however, sex was not considered as a biological variable. Enrollment took place between 18 September 2020 and 8 April 2021. The onset of COVID-19 symptoms needed to be within 7 days of randomization, and SARS-CoV-2 infection was confirmed by molecular diagnostic assay within 4 days of screening. Participants were excluded from the study if they had a previous hospitalization for COVID-19, had received previous treatment for COVID-19, had received a SARS-CoV-2 vaccine, or were receiving supplemental oxygen or hospital care at the time of screening.

Participants were randomized 1:1 to receive intravenous remdesivir (200 mg at baseline [Day 1] followed by 100 mg on Days 2 and 3) or matching placebo (Fig. 1). Nasopharyngeal swabs and sputum samples were collected at baseline and again on Days 2, 3, 7, and 14.

### Virologic resistance analyses

Samples from participants were eligible for virologic resistance analyses if their viral load was above the lower limit of quantification for the quantitative real-time polymerase chain reaction (PCR) viral load assay (2228 copies/mL). The whole genome of SARS-CoV-2 was sequenced. Amplicons were produced using the ARTIC primer set (42, 43), and the nucleotide sequence was determined by deep whole genome sequencing using Illumina MiSeq or NextSeq (DDL Diagnostic Laboratory, The Netherlands). Sequencing of SARS-CoV-2 samples collected at baseline, Day 3 (or Day 2 if viral load did not meet the sequencing threshold), Day 7, and Day 14 was conducted for all participants in the remdesivir group and 50% of those in the placebo group. SARS-CoV-2 lineage was determined by Pangolin software (v.3.1.11), and amino acid substitutions in Nsp12 at baseline were compared with the reference sequence (Wuhan-Hu-1, NC_045512.2). Postbaseline SARS-CoV-2 sequences were compared with baseline sequences for each individual participant for Nsp8, Nsp10, Nsp12, Nsp13, and Nsp14.

### Phenotypic analyses: subgenomic replicon system

The effect of emergent amino acid substitutions in SARS-CoV-2 Nsp8, Nsp10, Nsp12, Nsp13, and Nsp14 on the efficacy of remdesivir was assessed using site-directed mutagenesis in a SARS-CoV-2 subgenomic replicon system adapted and modified from previous studies (44, 45) and compared with the SARS-CoV-2 SH01 reference strain (SARS-CoV-2/human/CHN/SH01/2020, GenBank MT121215), which is a plaque-purified strain isolated from a patient in Shanghai and belongs to Pango lineage B. EC_50_ values were calculated as the concentration at which there was a 50% decrease in the luciferase reporter signal relative to vehicle (0% virus inhibition) and uninfected controls (100% virus inhibition). Results of the phenotypic analysis were reported as fold change in the effective concentration of remdesivir to reach EC_50_ relative to SH01. Any emergent Nsp12 substitutions were modeled on the cryo-electron microscopy structure of the SARS-CoV-2 replicase to determine the locations relative to the active site of Nsp12.

### Phenotypic analyses: recombinant infectious viral system

Recombinant SARS-CoV-2 expressing a luciferase reporter gene was generated using a previously described reverse genetic system (46). To introduce the intended substitutions into Nsp12, site-directed mutagenesis by PCR was performed on the pUC57-CoV-2-F4 plasmid using the Platinum SuperFi II DNA Polymerase (Invitrogen). Primers for site-directed mutagenesis of Nsp12 had the following sequences: A376V Forward GTATGCTGTTGACCCTGCTATGCACGC; A376V Reverse GCAGGGTCAACAGCATACACAAGTAATTCC; P323L Forward GTTCCCACTTACAAGTTTTG; P323L Reverse CAAAACTTGTAAGTGGGAAC. Following PCR, template DNA was digested with Anza DpnI (Invitrogen), followed by transformation into One Shot TOP10 Chemically Competent *E. coli* (Invitrogen). Following transformation, TACGen (Foster City, CA) picked individual clones for expansion, purified plasmid by Plasmid Maxi Kit (Qiagen, Hilden, Germany), and performed Sanger sequencing on viral sequences.

Wild-type or site-directed mutant pUC57-CoV-2-F4 was utilized for recombinant SARS-CoV-2 production, in combination with pCC1-CoV-2-F123 and pCC1-CoV-2-F567-FLuc or pCC1-CoV-2-F567-NLuc plasmids as described previously (46). Briefly, full-length cDNA was prepared by restriction enzyme digest, *in vitro* ligation, and purification with AMPure PB beads (Pacific Biosciences). Full-length and N-gene viral RNA were prepared by *in vitro* transcription using the mMESSAGE mMACHINE T7 transcription kit (Invitrogen) and purified using the MEGAclear transcription cleanup kit (Invitrogen). Twenty micrograms of N gene RNA and 20 µg of full-length viral RNA were electroporated into Vero-E6 cells expressing TMPRSS2. After electroporation, cells were evaluated daily for cytopathic effect. Viral RNA was isolated from Passage zero (P0) virus using the Direct-zol RNA MiniPrep kit (Zymo Research). Nsp12 was amplified from P0 viral RNA and from *in vitro* transcribed viral RNA using the SuperScript IV One-Step RT-PCR System (Invitrogen) to facilitate confirmatory sequencing of Nsp12 by TACGen.

### Replication capacity calculation

The replication capacity of the mutant replicon was calculated by dividing the luciferase signal of the mutant replicon by the wild-type replicon at 48 hours postinfection. Student’s *t*-test was performed using GraphPad Prism 8.1.2 to compare mutant and wild-type means.

### Expression and purification of SARS-CoV-2 RdRp complexes

To biochemically characterize the mechanisms of resistance, we expressed the Nsp7-Nsp8-Nsp12 complex using the baculovirus system. A pFastBac-1 vector (Invitrogen) was chemically synthesized (GenScript) with an insert containing the mutations of interest in the Wuhan-Hu-1 RdRp sequence (National Center for Biotechnology Information [NCBI]: QHD43415.1). Specifically, the polyprotein insert encodes for Nsp5 3CLpro, Nsp7, N-terminal 8 × polyhistidine-labeled Nsp8, and Nsp12 RdRp. Briefly, the polyprotein was expressed in Sf9 insect cells (Invitrogen) through the use of the MultiBac insect-cell expression system (Geneva Biotech) as previously described (18, 23). Protein complexes were purified by Ni-NTA affinity chromatography as per the manufacturer’s specifications (Thermo Fisher Scientific). The resulting protein sequences were validated by mass spectrometry (Dr. Jack. Moore, Alberta Proteomics and Mass Spectrometry).

### RNA synthesis assays

The enzymatic activity of the wild-type and mutant RdRps was determined as previously described (18, 23). Briefly, RdRps of interest were incubated at various concentrations in a 15 µL reaction containing 200 µM RNA primer, 2 µM RNA template (Dharmacon), 10 µM adenosine triphosphate, uridine triphosphate, and guanosine triphosphate (GE Healthcare) and 100 nM radiolabeled [α−32P] cytidine triphosphate (Perkin-Elmer) nucleotide for detection purposes. Reactions were incubated at 30°C and started with 5 mM MgCl_2_. Finally, reactions were stopped, boiled, and loaded on a 10% acrylamide-8M urea denaturing gel. The resulting RNA products were visualized by phosphorimaging.

## Data Availability

Full sequence data are publicly available in the GenBank database (accession no. PQ617276 to PQ618006). Data supporting the findings of this study are available from the corresponding author upon reasonable request.

## SUPPLEMENTAL MATERIAL

Supplemental material aac.01238-24-s0001.docx Tables S1 to S4.

ASM does not own the copyrights to Supplemental Material that may be linked to, or accessed through, an article. The authors have granted ASM a non-exclusive, world-wide license to publish the Supplemental Material files. Please contact the corresponding author directly for reuse.
