# Supplementary material for: SARS-CoV-2 resistance analyses from the Phase 3 PINETREE study of remdesivir treatment in nonhospitalized participants
Source: Antimicrob Agents Chemother. 2024 Dec 19;69(2):e01238-24. doi: 10.1128/aac.01238-24 (PMC11823660; doi:10.1128/aac.01238-24)
Supplement: Supplemental material — Tables S1 to S4. [file aac.01238-24-s0001.docx]

**SARS-CoV-2 resistance analyses from the Phase 3 PINETREE study of remdesivir treatment in nonhospitalized participants**

Lauren Rodriguez,^a^# Hery W. Lee,^b^ Jiani Li,^a^ Ross Martin,^a^ Dong Han,^a^ Simin Xu,^a^ Jasmine Moshiri,^a^ Nadine Peinovich,^a^ Gregory Camus,^a^ Jason K. Perry,^a^ Robert H. Hyland,^a^ Danielle P. Porter,^a^ Mazin Abdelghany,^a^ Matthias Götte,^b^ Charlotte Hedskog^a^

^a^Gilead Sciences, Inc., Foster City, CA, USA ^b^Department of Medical Microbiology and Immunology, University of Alberta, Edmonton, Canada.

**Running head:** SARS-CoV-2 resistance analyses of RDV in outpatients

#Address correspondence to Lauren Rodriguez, [lauren.rodriguez14@gilead.com](mailto:lauren.rodriguez14@gilead.com)

**Supplemental Material**

**Table S1. SARS-CoV-2 lineages at baseline**

| **Pango lineage (WHO nomenclature), n (%)^a^** | **Remdesivir  (N = 119)** | **Placebo  (N = 138)** | **Total  (N = 257)** |
| --- | --- | --- | --- |
| B.1.2 | 35 (29.4) | 43 (31.2) | 78 (30.4) |
| B.1.1.7 (Alpha) | 21 (17.6) | 27 (19.6) | 48 (18.7) |
| B.1.429 (Epsilon) | 9 (7.6) | 14 (10.1) | 23 (8.9) |
| B.1.243 | 8 (6.7) | 2 (1.4) | 10 (3.9) |
| B.1.427 | 4 (3.4) | 2 (1.4) | 6 (2.3) |
| B.1.234 | 2 (1.7) | 3 (2.2) | 5 (1.9) |
| B.1.311 | 1 (0.8) | 4 (2.9) | 5 (1.9) |
| B.1 | 0 | 4 (2.9) | 4 (1.6) |
| B.1.1.519 | 3 (2.5) | 1 (0.7) | 4 (1.6) |
| B.1.240 | 3 (2.5) | 1 (0.7) | 4 (1.6) |
| B.1.526 (Iota) | 2 (1.7) | 2 (1.4) | 4 (1.6) |
| B.1.561 | 1 (0.8) | 3 (2.2) | 4 (1.6) |
| B.1.577 | 3 (2.5) | 1 (0.7) | 4 (1.6) |
| B.1.595 | 2 (1.7) | 2 (1.4) | 4 (1.6) |
| B.1.596 | 0 | 4 (2.9) | 4 (1.6) |
| B.1.623 | 4 (3.3) | 0 | 4 (1.6) |
| Other^b^ | 22 (18.5) | 25 (18.1) | 47 (18.3) |

WHO, World Health Organization. ^a^Detected in ≥4 participants at baseline. ^b^Lineages observed in <4 participants included B.1.110.3, B.1.177, B.1.369, and P.1 (Gamma) observed in 3 participants each; B.1.177.12, B.1.564, B.1.582, B.1.637, and XB observed in 2 participants each; and A.2.5, B.1.1, B.1.1.135, B.1.1.192, B.1.1.231, B.1.1.316, B.1.1.328, B.1.1.337, B.1.1.517, B.1.111, B.1.160, B.1.170, B.1.177.21, B.1.232, B.1.241, B.1.265, B.1.280, B.1.396, B.1.438.4, B.1.568, B.1.575.1, B.1.609, C.23, Q.3, and R.1 observed in 1 participant each.

**Table S2. Emergent Nsp12 amino acid substitutions in participants with baseline and postbaseline sequencing data available**

| **Nsp12 substitutions detected postbaseline, n (%)** | **Remdesivir  (N = 115)** | **Placebo  (N = 129)** | **Total  (N = 244)** |
| --- | --- | --- | --- |
| S6L | 1 (0.9) | 0 | 1 (0.4) |
| G44V | 0 | 1 (0.8) | 1 (0.4) |
| K103M | 0 | 1 (0.8) | 1 (0.4) |
| E144G^a^ | 0 | 1 (0.8) | 1 (0.4) |
| D155H^a^ | 0 | 1 (0.8) | 1 (0.4) |
| T206I | 1 (0.9) | 0 | 1 (0.4) |
| P232L | 1 (0.9) | 0 | 1 (0.4) |
| A376V | 1 (0.9)^b^ | 0 | 1 (0.4) |
| T394M | 1 (0.9)^b^ | 0 | 1 (0.4) |
| P505S | 0 | 1 (0.8) | 1 (0.4) |
| A526S | 1 (0.9) | 0 | 1 (0.4) |
| R631G^a^ | 0 | 1 (0.8) | 1 (0.4) |
| A634S | 1 (0.9) | 0 | 1 (0.4) |
| S649* | 0 | 1 (0.8) | 1 (0.4) |
| F694Y | 1 (0.9) | 2 (1.6) | 3 (1.2) |
| S835L^a^ | 0 | 1 (0.8) | 1 (0.4) |

^a^One participant had emergent Nsp12 substitutions E144G, D155H, R631G, and S835L. ^b^One participant had a viral load >5 log_10_ copies/mL at Day 7. *Denotes translational termination(stop) codon.

**Table S3. Emergent Nsp8, Nsp10, Nsp13, and Nsp14 amino acid substitutions in participants with baseline and postbaseline sequencing data available**

| **Amino acid substitutions detected postbaseline, n (%)** | **Remdesivir  (N = 115)** | **Placebo  (N = 129)** | **Total  (N = 244)** |
| --- | --- | --- | --- |
| Nsp8 |  |  |  |
| S41F | 1 (0.9) | 0 | 1 (0.4) |
| T141M | 0 | 1 (0.8) | 1 (0.4) |
| Q186L | 0 | 1 (0.8) | 1 (0.4) |
| Nsp10 |  |  |  |
| A1D | 1 (0.9) | 0 | 1 (0.4) |
| S33I | 0 | 1 (0.8) | 1 (0.4) |
| H48Y | 0 | 1 (0.8) | 1 (0.4) |
| T102I | 2 (1.7) | 0 | 2 (0.8) |
| Nsp13 |  |  |  |
| P53L | 0 | 1 (0.8)^a^ | 1 (0.4) |
| T137M | 1 (0.9) | 0 | 1 (0.4) |
| G196V | 1 (0.9) | 0 | 1 (0.4) |
| E201K | 1 (0.9) | 1 (0.8)^a^ | 2 (0.8) |
| A208V | 0 | 1 (0.8) | 1 (0.4) |
| T228I | 0 | 1 (0.8)^a^ | 1 (0.4) |
| P238L | 0 | 1 (0.8)^a^ | 1 (0.4) |
| D260Y | 0 | 1 (0.8) | 1 (0.4) |
| E341D | 1 (0.9) | 0 | 1 (0.4) |
| S513L | 0 | 1 (0.8) | 1 (0.4) |
| T532I | 0 | 1 (0.8)^a^ | 1 (0.4) |
| S577F | 1 (0.9) | 0 | 1 (0.4) |
| A598V | 1 (0.9) | 0 | 1 (0.4) |
| Nsp14 |  |  |  |
| V182L | 0 | 1 (0.8) | 1 (0.4) |
| A214S | 1 (0.9) | 0 | 1 (0.4) |
| A274V | 0 | 1 (0.8)^b^ | 1 (0.4) |
| D331Y | 0 | 1 (0.8) | 1 (0.4) |
| P335S | 0 | 1 (0.8)^b^ | 1 (0.4) |
| V341L | 0 | 1 (0.8) | 1 (0.4) |
| K362R | 1 (0.9) | 0 | 1 (0.4) |
| L409F | 0 | 1 (0.8) | 1 (0.4) |
| E453D | 0 | 1 (0.8) | 1 (0.4) |
| Y517S | 0 | 1 (0.8)^b^ | 1 (0.4) |

^a^One participant had emergent Nsp13 substitutions E201K, T228I, P53L, P238L, and T532I. ^b^One participant had emergent Nsp14 substitutions A274A/V, P335P/S, Q343Q/*, and Y517Y/S.

**Table S4. Attempts to regenerate recombinant SARS-CoV-2 encoding Nsp12 A376V substitution via a ligation-based reverse-genetics system**

| **Virus rescue** | **Nsp12 position 376 amino acid** | **Viral background sequence** | **Reporter gene** |
| --- | --- | --- | --- |
| + | Alanine (WT) | WA1 | Firefly luciferase |
| – | Valine | WA1 | Firefly luciferase |
| + | Alanine (WT) | WA1 | Nanoluciferase |
| Revert to WT | Valine | WA1 | Nanoluciferase |
| + | Alanine (WT) | WA1 + Nsp12 P323L | Nanoluciferase |
| – | Valine | WA1 + Nsp12 P323L | Nanoluciferase |

WA1, Washington-1 SARS-CoV-2 isolate; WT, wild type. + denotes successful rescue; – denotes no viral RNA or cytopathic effect detected
